# Diabetes and mortality in patients with prostate cancer: a meta-analysis

**DOI:** 10.1186/s40064-016-3233-y

**Published:** 2016-09-13

**Authors:** Junga Lee, Edward Giovannucci, Justin Y. Jeon

**Affiliations:** 1 Department of Sport and Leisure Studies, Yonsei University, Seoul, South Korea; 2 Exercise Medicine Center for Diabetes and Cancer Patients, Yonsei University, Seoul, South Korea; 3 Departments of Nutrition and Epidemiology, Harvard School of Public Health, Boston, MA USA

**Keywords:** Prostate cancer, Diabetes, All-cause mortality, Prostate cancer-specific mortality, Type 2 diabetes

## Abstract

**Background:**

There are conflicting results as to the association between pre-existing diabetes and the risk of mortality in patients with prostate cancer. The purpose of this study is to estimate the influence of pre-existing diabetes on prostate cancer-specific mortality and all-cause mortality.

**Methods:**

We searched PubMed and Embase to identify studies that investigated the association between pre-existing diabetes and risk of death among men with prostate cancer. Pooled risk estimates and 95 % confidence intervals were calculated using fixed-effects models or random-effects models. Heterogeneity tests were conducted between studies. Publication bias was analyzed by using the *Egger’s test*, *Begg’s test*, and the trim and fill method.

**Results:**

Of the 733 articles identified, 17 cohort studies that had 274,677 male patients were included in this meta-analysis. Pre-existing diabetes was associated with a 29 % increase in prostate cancer-specific mortality [relative risk (RR) 1.29, 95 % CI 1.22–1.38, I^2^ = 66.68 %], and with a 37 % increase in all-cause mortality (RR 1.37, 95 % CI 1.29–1.45, p < 0.01, I^2^ = 90.26 %). Additionally, in a subgroup analysis that was a type specific analysis focusing on type 2 diabetes and was conducted only with three cohort studies, pre-existing type 2 diabetes was associated with all-cause mortality (RR 2.01, 95 % CI 1.37–2.96, I^2^ = 95.55 %) and no significant association with prostate cancer-specific mortality was detected (RR 1.17, 95 % CI 0.96–1.42, I^2^ = 75.59 %). There was significant heterogeneity between studies and no publication bias was found.

**Conclusions:**

This meta-analysis suggests diabetes may result in a worse prognosis for men with prostate cancer. Considering heterogeneity between studies, additional studies should be conducted to confirm these findings, and to allow generalization regarding the influence that each type of diabetes has on prostate cancer mortality.

## Background

Prostate cancer is the second leading cause of cancer death among men in the United States (Siegel et al. 2014). In 2015, the American Cancer Society reported that 1 in 7 men will be diagnosed with prostate cancer during his lifetime (Jemal et al. 2011). The known risk factors for prostate cancer include age, ethnicity, and family history (Jemal et al. 2011). Diabetes mellitus influences the risk of various cancers, including colon, pancreas, and thyroid cancer (Giovannucci et al. 2007; Karlin et al. 2012; Kasper et al. 2009). Prostate cancer appears to be an exception, whereby a diagnosis of diabetes is associated with a reduced incidence in most studies. However, whether a previous history of diabetes influences the prognosis of prostate cancer is not clear (Batty et al. 2011; Bensimon et al. 2014; Fleming et al. 2003; Froehner et al. 2003; Smith et al. 2008; Yeh et al. 2012).

Diabetes is primarily divided into type 1 and type 2 diabetes. Recently, the prevalence of type 2 diabetes has rapidly escalated owing to the increase in obesity, which also increases the risk of various cancers (Baba et al. 2011). Although hyperinsulinemia was hypothesized as the link between type 2 diabetes and the risk of various cancers (Barone et al. 2008), the influence of diabetes on the risk and prognosis of prostate cancer is complex because of the testosterone fluctuations often observed among patients with diabetes, and these fluctuations also influence the development of prostate tumors (Baradaran et al. 2009).

Pre-existing diabetes is defined as having a diagnosis of diabetes before the prostate cancer was diagnosed. Some prospective cohort studies reported that pre-existing diabetes was associated with 32 % increased risk of mortality among prostate cancer patients (Liu et al. 2012), while other studies reported that pre-existing diabetes was not associated with the prognosis of prostate cancer (Chiou et al. 2012). Since here is a controversy whether pre-existing diabetes would affect prognosis of prostate cancer, a meta-analysis would be necessary. There was two prior meta-analyses have studied association between pre-existing diabetes and prognosis of prostate cancer. These two studies indicated that pre-existing diabetes was associated with increased risk of prostate cancer death (Cai et al. 2015; Snyder et al. 2010). However, the meta-analysis by Snyder et al. only included four cohort studies (Snyder et al. 2010), while Cai et al. included 11 cohort studies (Cai et al. 2015). Since these meta-analyses, there are six additional cohort studies, which further examine the association between pre-existing diabetes and prognosis of prostate cancer. By including more studies, it is now possible to further examine subgroup analyses such as types of diabetes, level of adiposity and country where study was conducted. Therefore, we have included 17 cohort studies, which met our inclusion criteria to examine association between pre-existing diabetes and prognosis of prostate cancer.

## Methods

### Data sources and searches

This meta-analysis study followed the *Preferred Reporting Items for Systematic Reviews and Meta*-*Analyses (PRISMA)* guidelines. The analysis used the MEDLINE and EMBASE databases to identify applicable studies that were published between January 1970 and August 2016. The studies selected for inclusion needed to evaluate the effects of diabetes mellitus on the risk of death in patients with prostate cancer and they should have been published in the English language and in peer-reviewed journals. The search terms used in this study were “diabetes mellitus”, “prostate cancer”, “survival”, “prognosis”, “death”, and “mortality”. After a study was considered relevant on the basis of the search terms, its references were manually examined to find additional relevant studies. This study selected articles that reported finding in two categories: (1) the association of diabetes with prostate cancer-specific mortality in prostate cancer patients, and (2) the association of diabetes with all-cause mortality in prostate cancer patients. We then separately pooled the results from these two categories, to determine the relationship between type 2 diabetes and both prostate cancer-specific mortality and all-cause mortality, exclusively among prostate cancer patients. Pre-existing diabetes is defined as having a diagnosis of diabetes before the prostate cancer was diagnosed.

### Eligibility criteria

Two authors (JL and JYJ) independently reviewed the articles in a standardized manner. Any disagreements in the eligibility for study selection were discussed by all three authors (JL, JYJ, and EG) to obtain a consensus. To be included in this study, each study had to meet three criteria: (1) evaluate prostate cancer, (2) indicate ascertainments of diabetes, including self-report, medication use, and blood test, and (3) report the hazard ratio or relative risk using standard error or a 95 % confidence interval (CI). In cases of publications that were duplicated or originated from the same study population, only the most recent study with the longest follow-up duration was included.

### Data extraction and quality assessment

Two authors (JL and JYJ) evaluated the selected articles by following the guidelines of the Meta-analysis of Observational Studies in Epidemiology (MOOSE). In case of discrepancies, all three authors (JL, JYJ, and EG) conducted further discussions to obtain a consensus. The following data elements were extracted for this meta-analysis study: last name of the first author, publication year, country where the study was performed, number of deaths, sample size, description of the method used to diagnose diabetes, outcome determination, age at baseline, adjustment factors, follow-up duration, criteria of the cause of death, and the relative risk or hazard ratio that corresponded to a 95 % CI.

The authors evaluated the quality of the selected studies using the Newcastle-Ottawa Scale for the following factors: clarification as to diabetes status, adjustment for intermediate factors (e.g., age, disease stage, and tumor differentiation), study endpoints for prostate cancer-specific mortality and all-cause mortality, duration of follow-up, representativeness of the exposed cohort, and adequacy of the follow-up of cohorts (Table 1).

**Table 1:** Diabetes and mortality in prostate cancer

First author (year), name of study, country	Sample characteristics (sample size, study recruitment period)	Follow-up period (year), study design	Criteria of the cause of death	Comparisons	RR (95 % CI)				Adjustment factors
Park (2006), National Health Insurance Corporation Study and Korean Central Cancer Registry, South Korea	256 men (1996–2004)	Median: 3.03 years, prospective cohort study	National statistical data	Fasting serum glucose <110	Prostate cancer-specific mortality 1.81 (0.61–5.40)				Age, alcohol consumption, BMI, cholesterol level, physical activity, food preference, blood pressure, and other comorbidities
Merrick (2007), Schiffler Cancer Center, USA	530 men (1995–2003)	Median: 5.7 years, prospective cohort study	Documentations for cause of death	Diabetes versus non-diabetes	Prostate cancer-specific mortality 2.41 (1.14–5.15)				Smoking, age, percent of positive biopsies, and body mass index
Van de Poll-Franse (2007), Eindhoven Cancer Registry, the Netherlands	5478 men (1995–2002)	3–10 years, prospective cohort study	Documentations for cause of death	Diabetes versus non-diabetes	All-cause mortality 1.19 (1.04–1.37)				Age, disease stage, treatment, and cardiovascular disease
Smith (2008), Radiation Therapy Oncology Group Protocol 92–02, USA	1551 men (1992–1995)	Median: 8.17 years, prospective cohort study	Documentations for cause of death	Diabetes versus non-diabetes	All-cause mortality 1.77 (1.45–2.16) Prostate cancer mortality 0.80 (0.51–1.25) Non-prostate cancer-specific mortality 2.12 (1.69–2.66)				Age, ethnicity, tumor stage, Gleason score, prostate-specific antigen, weight, and treatment arm
D’Amico (2010), Chicago Prostate Cancer Center, USA	5279 men (1997–2007)	Median: 3.9 years, prospective cohort study	Documentations for cause of death	Diabetes versus non-diabetes	Prostate cancer-specific mortality 1.28 (0.54–3.03) Non-prostate cause-specific mortality 1.53 (1.13–2.07)				History of myocardial infraction, treatment received, age, year of brachytherapy, and prostate cancer risk group
Tseng (2011), Department of Health, Executive Yuan, Taiwan	102,651 men (1995–2006)	12 years, prospective cohort study	ICD-9	Type 2 diabetes versus non-diabetes	All-cause mortality (diabetes) Diabetes of any duration at enrollment				–
					Age	n	N	RR	
					40–64	17	23,958	6.72 (4.43–10.19)	
					65–74	58	12,395	2.76 (2.15–3.55)	
					≥ 75	33	3574	1.51 (1.08–2.13)	
					All-cause mortality (type 2 diabetes) Diabetes of any duration at enrollment				
					Age	n	N	RR	
					40–64	16	23,197	6.52 (4.24–10.03)	
					65–74	58	12,051	2.74 (2.12–3.53)	
					≥ 75	53	3448	1.56 (1.11–2.19)	
Batty (2011), Whitehall Study, London, UK	17,934 men (1967–1970) 40–69 years of age	Maximum: 40 years, prospective cohort study	ICD8/9:185, ICD10:C61	Diabetes versus non-diabetes	Prostate cancer-specific mortality 0.24 (0.03–1.73)				BMI, plasma cholesterol, physical activity, socio-economic status, diabetes/blood glucose, marital status, FEV1, height, smoking, diastolic and systolic blood pressure, and age at risk
Chamie (2012), Greater Los Angeles and Long Beach Veterans Affairs Medical Center, USA	1031 men (1997–2004), 66–75 years of age	10 years, prospective cohort study	Social security death index	Diabetes versus non-diabetes	Prostate cancer-specific mortality Diabetes without end-organ damage 2.32 (1.32–4.08) Diabetes with end-organ damage 4.27 (1.64–11.10)				–
Chiou (2012), Chang Gung Memorial Hospital in Linkou, Taiwan	81,564 men (2001–2010)	9 years, prospective cohort study	ICD-9	Type 2 diabetes versus non-diabetes	Prostate cancer-specific mortality Non-diabetes 0.47 (0.38–0.59) Diabetes 0.82 (0.59–1.13)				Age
Liu (2012), Center for Primary Health Care Research, Sweden	2217 men (1961–2008)	7 years, prospective cohort study	ICD-9	Type 2 diabetes versus non-type 2 diabetes	Prostate cancer-specific mortality 1.32 (1.23–1.41)				Age at diagnosis, diabetes period, obesity, alcohol, smoking, socioeconomic status, and diagnosis site
Karlin (2012), Academic Medical Center located in metropolitan USA	4347 men (1999–2008)	Median: 4 prospective cohort study. 5 years	ICD-9	Diabetes versus non-diabetes	All-cause mortality 1.36 (1.05–1.76)				Age
Currie (2012), A retrospective cohort study, U.K.	15,951 men (1990–2009)	Mean: 6.7 (± 0.08) years, Retrospective cohort study Median: 9.3 years (9.2–9.4)	Documentations for cause of death	Type 2 diabetes versus non-diabetes	All-cause mortality 1.19 (1.08–1.31)				Age at baseline, smoking history, Charleston comorbidity index, and year of diagnosis
Shetti (2012), American Joint Committee on Cancer, USA	1624 men (1995–2006)	Mean: 7.8 years Median: 7.6 years	Documentations for cause of death	Diabetes versus non-diabetes	All-cause mortality 1.54 (1.10–2.15)				Age, PSA, Gleason score, percent positive biopsies, BMI, prostate volume, clinical stage, XRT, ADT, ADT duration, perennial invasion, hypertension, hypercholesterolemia, CAD, and tobacco use
Yeh (2012), a local campaign against cancer and heart disease (CLUE II) cohort study, USA	18,280 men (1989–2006)	17 years, prospective cohort study	National death Index, Maryland death	Diabetes versus non-diabetes	All-cause mortality 1.43 (0.31–6.69)				Age, sex, BMI, smoking, education level, hypertension treatment, and high cholesterol treatment
Bensimon (2014), National Cancer Date Repository (NCDR), Clinical Practice Research Datalink (CPRD), Hospital Episode Statistics (HES) database, and Office for National Statistics (ONS) database, UK	11,920 men (1998–2012)	Mean: 4.7 (±0.08) years, prospective cohort study	Documentations for cause of death	Type 2 diabetes versus non-type 2 diabetes	Prostate cancer-specific mortality 1.23 (1.04–1.46) All-cause mortality 1.25 (1.11–1.40)				Age, year of cohort entry, ethnicity, excessive alcohol use, BMI, smoking status, chronic kidney disease, myocardial infarction, ischemic stroke, transient ischemic attack, peripheral artery disease, previous cancer, angiotensin-converting enzyme inhibitors, angiotensin receptor blockers, calcium channel blockers, beta-blockers, diuretics, other antihypertensive drugs, aspirin, other nonsteroidal anti-inflammatory drugs, statins, 5-alpha reductive inhibitors, and the following prostate cancer-related variables: PSA levels, Gleason score, radical prostatectomy, radiation therapy, chemotherapy, and ADT
Best (2015), Strong Heart Study, USA	1784 men (1989–1991)	17.2 years, prospective cohort study	ICD9	Diabetes versus non-diabetes	Prostate cancer-specific mortality 2.96 (1.15–7.57)				Age, stratified by center, BMI, Education, drinking status, and smoking status
Polesel (2016), Multicentre hospital-based-control study	715 men (1995–2002)	11.6 years Retrospective cohort study	Regional health care system databases	Diabetes versus non-diabetes	Prostate cancer-specific mortality 0.64 (0.22–1.88) All-cause mortality 1.56 (1.03–2.36)				Age at diagnosis, years of education, Gleason score, and smoking

*ADT* androgen deprivation therapy, *BMI* body mass index, *CAD* coronary artery disease, *FEV1* forced expiratory volume in 1 s, *PSA* prostate specific antigen, *XRT* X-radiation therapy, *ICD* international classification of diseases

### Statistical analysis

This meta-analysis study combined the risk estimates with CI or SE to estimate prostate cancer-specific mortality and all-cause mortality. The statistical heterogeneity between studies was estimated using Q statistic, and inconsistency was quantified using the *I*^2^ statistic (Borenstein et al. 2005). Fixed-effect models with forest plots were used to pool the results of homogeneous studies whereas random-effect models with forest plots were used for heterogeneous studies.

Publication bias was evaluated using the Egger test (Egger et al. 1997) and Begg’s test (Begg and Mazumdar 1994). To further assess the potential effects of publication bias, the Duval and Tweedie nonparametric trim and fill method was used (Duval and Tweedie 2000). This method considers the possibility of hypothetically missing studies, imputes their RRs, and then recalculates a pooled estimate (Borenstein et al. 2010). Statistical significance was estimated using a p value of <0.05. All statistical analyses were performed using the Comprehensive Meta-Analysis software version 1.25 (Biostatic, Inc., Englewood, NJ, USA).

## Results

### Literature search

**Fig. 1:**
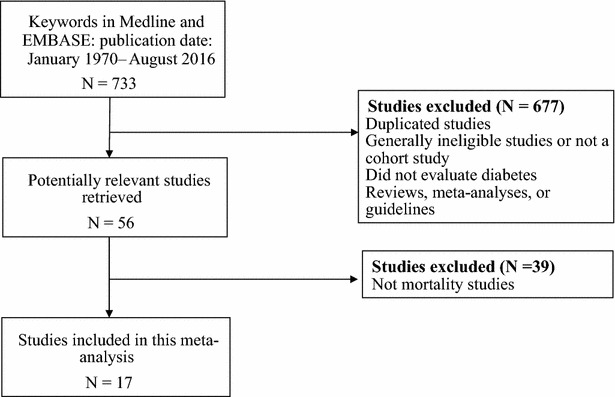
Flow diagram of the process for selecting studies for this meta-analysis

This meta-analysis study followed the selection processes shown in Fig. 1, by using the above-discussed exclusion and inclusion criteria. Of the 733 searched studies initially identified, 677 were excluded for the following reasons: presented duplicate information, did not report prostate cancer-specific mortality or all-cause mortality, were reviews or meta-analyses, or did not evaluate diabetes mellitus. An additional 39 studies were excluded from this analysis, because they were not mortality studies that evaluated diabetes. After applying the selection criteria, only 17 studies were included (Table 1). The total number of patients with prostate cancer was 274,677. The follow-up periods ranged between 3 and 17 years. This meta-analysis pooled directly the relative risk of prostate cancer-specific mortality and all-cause mortality from the 17 selected studies and then calculated the overall prostate cancer-specific and all-cause mortality, respectively. This study included only prior studies that had prospective and retrospective cohort designs, in order to understand the association between pre-existing diabetes and the prospect of prostate cancer mortality.

### Study characteristics

A summary of the descriptive data is presented in Table 1. The majority of the studies selected for this meta-analysis were conducted in the United States (Best et al. 2015; Chamie et al. 2012; D’Amico et al. 2010; Karlin et al. 2012; Merrick et al. 2007; Shetti et al. 2012; Smith et al. 2008; Yeh et al. 2012). The remaining studies were conducted in the Republic of Korea (Park et al. 2006), Sweden (Liu et al. 2012), Netherlands (van de Poll-Franse et al. 2007), United Kingdom (Batty et al. 2011; Bensimon et al. 2014; Currie et al. 2012), Italy (Polesel et al. 2016) and Taiwan (Chiou et al. 2012; Tseng 2011). All 17 studies were published within the last 10 years.

In the studies selected, the prevalence of pre-existing diabetes ranged between 18 and 24 %. The average age of the participants was 58 years. The methods used for determining the existence of diabetes were: (1) medical records; (2) documented use of diabetes medicine; (3) the International Classification of Diseases (ninth revision) diagnosis codes for surveillance, epidemiology, and end results (Medicare); or (4) fasting glucose level. Most studies used in the meta-analysis adjusted for age and for other factors, including disease stage, alcohol use, smoking history, and physical activity (Table 1).

### Association between diabetes and mortality

**Fig. 2:**
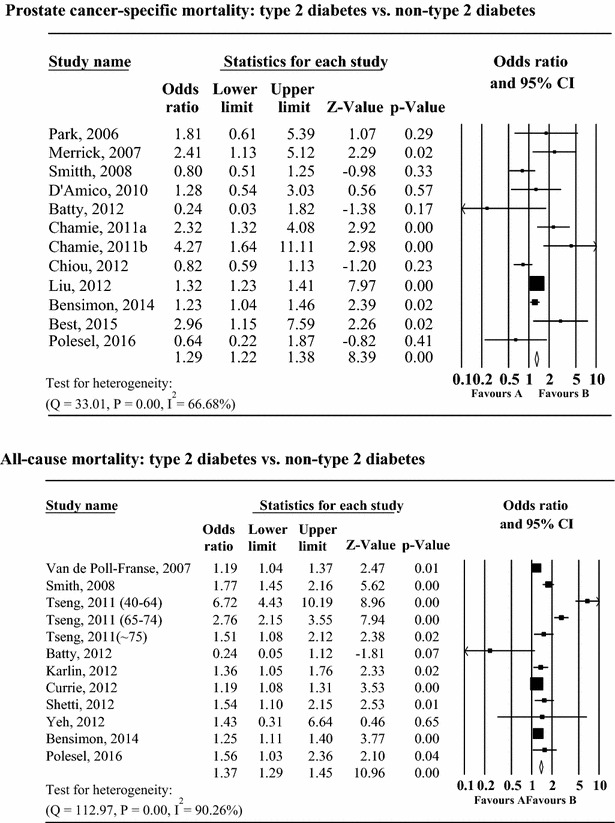
Relative risk for the association between pre-existing diabetes, prostate cancer-specific mortality, and all-cause mortality

Seventeen studies examined the association between pre-existing diabetes, prostate cancer-specific mortality, and all-cause mortality in patients with prostate cancer (Fig. 2). The association between pre-existing diabetes and the risk of prostate cancer-specific mortality indicated that pre-existing diabetes was significantly associated with a 30 % increase in the risk of prostate cancer-specific mortality (RR 1.29, 95 % CI 1.22–1.38, I^2^ = 66.68 %, p < 0.01) and with a 65 % increase in the risk of all-cause mortality (RR 1.37, 95 % CI 1.29–1.45, p < 0.01, I^2^ = 90.26 %, p < 0.01). There was significant heterogeneity between studies. There was no evidence of publication bias on the basis of analyses using the Egger test and Begg’s test. In addition, there was no influence of unpublished data in any analysis using the trim and fill method.

### Association between type 2 diabetes and mortality in a subgroup analysis

**Fig. 3:**
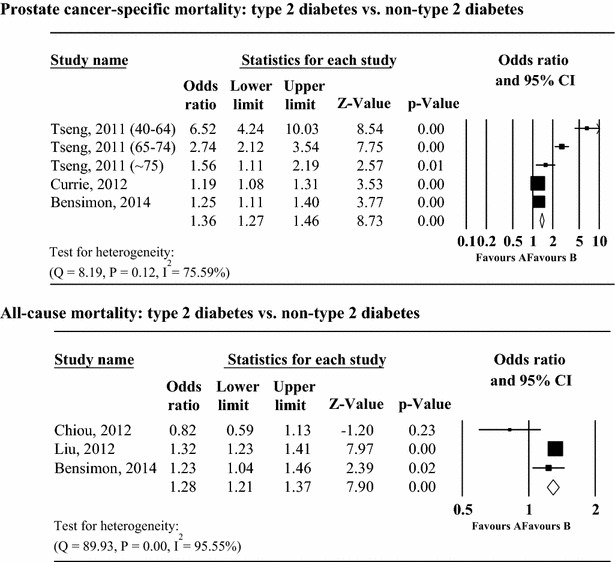
Relative risk for the association between pre-existing type 2 diabetes, prostate cancer-specific mortality, and all-cause mortality

To further examine whether pre-existing type 2 diabetes, separately from type 1 diabetes, was associated with the prognosis of prostate cancer, five studies, which evaluated only type 2 diabetes, were included in this ancillary analysis (Fig. 3). This analysis showed that pre-existing type 2 diabetes was significantly associated with all-cause mortality (RR 2.01; 95 % CI, 1.37–2.96, I^2^ = 75.59 %, p < 0.01) whereas no association was found between pre-existing type 2 diabetes and prostate cancer-specific mortality. Significant heterogeneity was found between studies. In addition, there was no evidence of publication bias using the Egger test and Begg’s test and unpublished data were not found in any analysis using the trim and fill method.

## Discussion

This meta-analysis study evaluated the association of pre-existing diabetes on prostate cancer-specific mortality and all-cause mortality. A main finding of this meta-analysis was that prostate cancer patients with pre-existing diabetes had an approximately 29 % higher prostate cancer-specific mortality and approximately 37 % higher all-cause mortality. In our ancillary analysis, which only included results from patients with pre-existing type 2 diabetes, we found that patients with prostate cancer with type 2 diabetes had a doubling in all-cause mortality. Although we are cautious in asserting that pre-existing diabetes is a key causal factor for worse prognosis of prostate cancer, taking preventative measures towards precluding diabetes is appropriate for patients with prostate cancer.

This meta-analysis included 17 studies. Of these, 11 studies addressed prostate cancer-specific mortality and 10 addressed all-cause mortality. Two previous meta-analyses reported the association between pre-existing diabetes and prognosis of prostate cancer (Begg and Mazumdar 1994; Liu et al. 2012). The meta-analysis by Snyder et al. (2010) of four cohort studies indicated that patients with prostate cancer with pre-existing diabetes had a 57 % higher all-cause mortality, whereas the meta-analysis by Cai et al. (2015) of 11 studies indicated that patients with prostate cancer with pre-existing diabetes had a 26 % higher prostate cancer-specific mortality and 83 % higher non-prostate cancer mortality. Current meta-analysis found six additional studies that have reported the impact of pre-existing diabetes on prognosis of prostate cancer (Chamie et al. 2012; Chiou et al. 2012; Giovannucci and Chan 2010; Karlin et al. 2012; Liu et al. 2012; Tseng et al. 2012), and the data available in the literature enabled us to perform an ancillary analysis using the studies that evaluated only patients with type 2 diabetes. Our analysis showed that type 2 diabetes increased the all-cause mortality by approximately 100 % when compared to patients with prostate cancer without diabetes. The results from our and other meta-analyses clearly showed that pre-existing diabetes, whether type 2 diabetes alone or both type 1 and 2 diabetes, increased the risk of all-cause mortality in patients with prostate cancer. The association between type 2 diabetes and prostate cancer mortality was not significant compared to the significant association seen for total diabetes mellitus, but it might be related to the sub-group analysis between type 2 diabetes and prostate cancer mortality was conducted only with 3 studies. Also, in assessing the observed positive results from this, there must be an acknowledgement that prostate cancer patients with diabetes have been found not do well with their diabetes treatment/management as well as their anti-cancer treatment/management. Unfortunately, our selected studies did not report the results after an adjustment for diabetes and cancer treatment/management. A further study that would reflect adjustment for this issue could help towards a better understanding of the relationship between diabetes and cancer treatment/management.

There have been controversies about whether pre-existing diabetes is associated with the incidence and prognosis of prostate cancer (Bensimon et al. 2014; D’Amico et al. 2010; Liu et al. 2012; Tseng 2011). Some studies (Batty et al. 2011; Chiou et al. 2012; Smith et al. 2008) suggested an inverse association, but with very wide CI’s. These studies argued that lower androgen levels in patients with type 2 diabetes contributed to the better prognosis of prostate cancer. However, other studies showed significantly worse prognosis in patients with prostate cancer with diabetes. It is not clear as to why some studies found an inverse association whereas others found a direct association between diabetes and prognosis of prostate cancer. Our meta-analyses demonstrated that pre-existing diabetes is associated with worse prognosis of prostate cancer.

Worse prognosis in patients with prostate cancer with diabetes may be related to several mechanisms. First, patients with prostate cancer with diabetes are more likely to have progressive prostate cancer due to the adverse interaction between diabetes mellitus and prostate cancer (Lubik et al. 2011; Ma et al. 2008). Patients with diabetes present with hyperglycemia, and these factors are associated with tumor development and progression (de Beer and Liebenberg 2014; Lai et al. 2014; Venkateswaran et al. 2007). Second, diabetes can diminish the effects of radiotherapy on prostate cancer. Accordingly, patients with prostate cancer with diabetes are more likely to experience a higher failure rate of radiotherapy treatment and worse gastrointestinal and genitourinary complications compared to patients with prostate cancer without diabetes (Chan et al. 2005; Herold et al. 1999). These complications can be explained by possible alterations in insulin-like growth factor 1, which may decrease the effectiveness of the treatments (Casa et al. 2008). Third, changes in certain hormones, including testosterone, sex hormone-binding globulin, and leptin, may affect the risk of prostate cancer (Baradaran et al. 2009). Additionally, previous studies (Basaria et al. 2006; Smith et al. 2006) indicated that low levels of androgens in patients with prostate cancer with diabetes contributed to insulin resistance as well as the risk of prostate cancer death and of non-prostate cancer death. In this respect, long-term androgen deprivation therapy, which is commonly used to treat patients with prostate cancer, increases insulin resistance and hyperglycemia, which in turn induces cardiovascular diseases (Basaria et al. 2006; Smith et al. 2006).

There are several limitations of this study. First, the duration of diabetes in the selected studies was not consistent. The duration of diabetes is crucial because recent findings indicate that longer durations of diabetes were associated with a higher risk of prostate cancer mortality and all-cause mortality (Bensimon et al. 2014). Second, the selected studies used different adjustment factors, such as tumor stage, treatment methods, and varying durations of diabetes, and these different adjustment factors may influence the RRs found in this study. Third, this meta-analysis study did not have adjustment for immortal time bias. This meta-analysis study was unable to address this issue as the prospective studies contained no information regarding follow up status for those men free of diabetes till time of diabetes diagnosis, and then till prostate cancer death occurred. Finally, the selected studies in this meta-analysis did not provide the Gleason scoring content, and therefore, we cannot rule out the possibility that only the high grade tumors are positively associated with diabetes, while the majority of prostate cancer tumors with low histologic grading may not be associated with diabetes co-morbidity.

This study suggested that pre-existing diabetes is clearly associated with total mortality and possibly prostate cancer-specific mortality in men diagnosed with prostate cancer. Future studies are necessary to select adequate treatments for patients with prostate cancer with diabetes in order to improve prognosis and reduce complications. In addition, these studies should examine the differences between type 1 and type 2 diabetes and determine how factors such as the duration of diabetes, radiotherapy treatments, and tumor stages can affect prostate cancer mortality.
